# Nanopore-based analysis of biochemical species

**DOI:** 10.1007/s00604-015-1560-2

**Published:** 2015-07-25

**Authors:** Nannan Liu, Zekun Yang, Xiaowen Ou, Benmei Wei, Juntao Zhang, Yongmei Jia, Fan Xia

**Affiliations:** Key Laboratory for Large-Format Battery Materials and Systems, Ministry of Education, School of Chemistry and Chemical Engineering, Huazhong University of Science and Technology (HUST), Wuhan, 430074 China; National Engineering Research Center for Nanomedicine, Huazhong University of Science and Technology, Wuhan, 430074 China

**Keywords:** Nanochannels, Electrochemical analysis, Ion channels, Nucleic acid analysis, Protein analysis, Sequencing

## Abstract

Biological nanochannels or nanopores play a crucial role in basic biochemical processes in cells. Artificial nanopores possessing dimensions comparable to the size of biological molecules and mimicking the function of biological ion channels are of particular interest with respect to the design of biosensors with a sensitivity that can go down to the fM level and even to single molecule detection. Nanopore-based analysis (NPA) is currently a new research field with fascinating prospects. This review (with 118 refs.) summarizes the progress made in this field in the recent 10 years. Following an introduction into the fundamentals of NPA, we demonstrate its potential by describing selected methods for sensing (a) proteins such as streptavidin, certain antibodies, or thrombin via aptamers; (b) oligomers, larger nucleic acids, or micro-RNA; (c) small molecules, (d) ions such as K(I) which is vital to the maintenance of life, or Hg(II) which is dangerous to health. We summarize the results and discuss the merits and limitations of the various methods at last.

**Graphical abstract:**
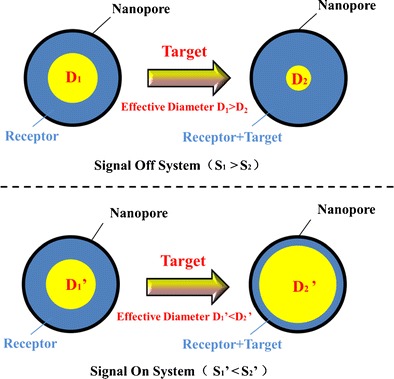
Schematic of a signal-off system and a signal-on system in nanopore analysis. The effective diameter of nanopores decreases when targets undergo certain interactions with receptors attached on the inner surface of the nanopore. Correspondingly, the current will drop on appearance of the analyte. This is referred to as a “signal-off” system. Conversely, it is called a “signal-on” system.

Schematic of a signal-off system and a signal-on system in nanopore analysis. The effective diameter of nanopores decreases when targets undergo certain interactions with receptors attached on the inner surface of the nanopore. Correspondingly, the current will drop on appearance of the analyte. This is referred to as a “signal-off” system. Conversely, it is called a “signal-on” system.

## Introduction

Biological ion channels embed in biological cell to communicate the matter and energy with the extracellular world [1]. They play a crucial role in various significant physiological activities [2–4]. The function of biological ion channels has allured a lot of attention from both scientists and engineers. They fabricate solid-state artificial nanopores to mimic functions of biological ion channels [5–9]. Except for many similar functions of the biological ion channels, synthetic nanopores such as carbon nanotubes [10–13], silicon-based nanopores [14–20], graphene nanopores [21–24], and polymeric nanopores [25–27] possess ascendances, multi-functions and stability. Benefit by these characteristics, many applications including sensing [28–32], energy conversion [33–35], nanofluidic circuits [36, 37] and filtration [38–40] are possible to achieve. Among all these potential applications, we focus mainly on the nanopore-based analysis (NPA) for their applications in detection of the biochemical species in last 10 years.

**Fig. 1:**
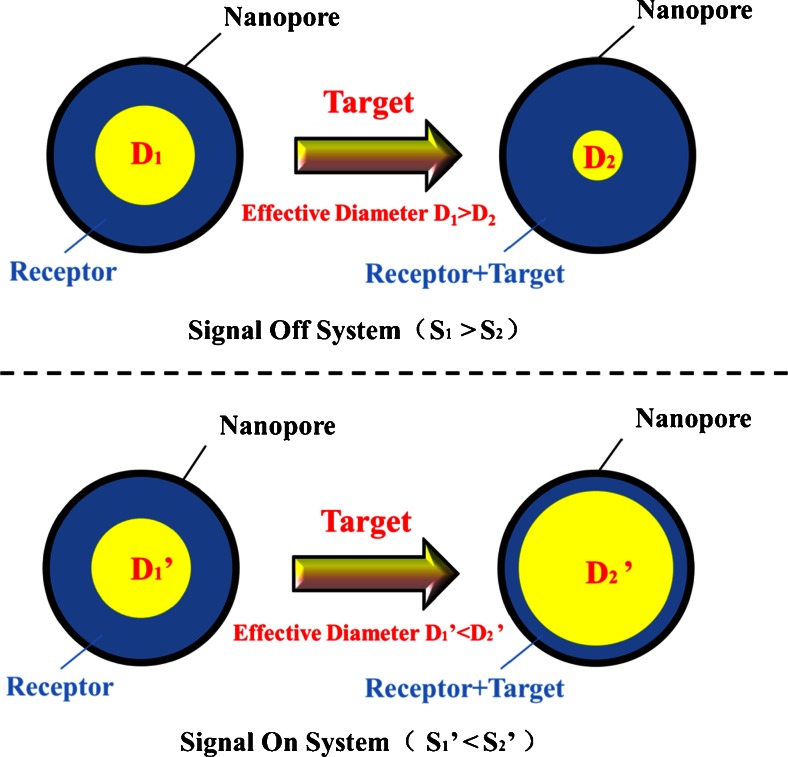
Schematic of the signal off system and signal on system illustrates the principle of the NPA. The effective diameter of nanopores will be decreased when targets have certain interaction with receptors which are attached on the inner surface of the nanopore. Correspondingly, the current signal will drop along with the appearance of the target; we call it signal off system. Conversely, we call it signal on system

The principle of NPA can be described briefly: molecules access in or attach on the surface of a pore, thereby leading the ionic current changes can be detected [41]. The nanopore membrane is located in the middle of two electrochemical chambers separated into cis- and trans- compartments, each containing conducting buffers. Under an applied voltage, electrolyte ions flow through the nanopore, which is measured as current in the electrical instrument. The effective diameter of the nanopore will be decreased when targets have certain interaction with receptors which are attached on the inner surface of the nanopore. Correspondingly, the current signal will drop along with the appearance of the target; we call it signal off system. Conversely, the effective diameter of nanopores will be increased when targets change the structure of receptor or come out of the nanopore. Correspondingly, the current signal will rise along with the appearance of the target; we call it signal on system (Fig. 1). Using nanopores in sensing of biomolecules has distinct advantages; for example, one can detect analytes via their size [42], shape [43] or charge [44]. The range of analytes that can be detected with nanopores now spans peptides, proteins, bimolecular complexes, enzymes, organic polymers and small molecules [45]. In this review, we discuss the application of the NPA in detection of nucleic acids, proteins, small molecules and ions. We end with a brief conclusion of the advantages and challenges of the NPA.

## The NPA for detection of biochemical species

### Detection of proteins

**Fig. 2:**
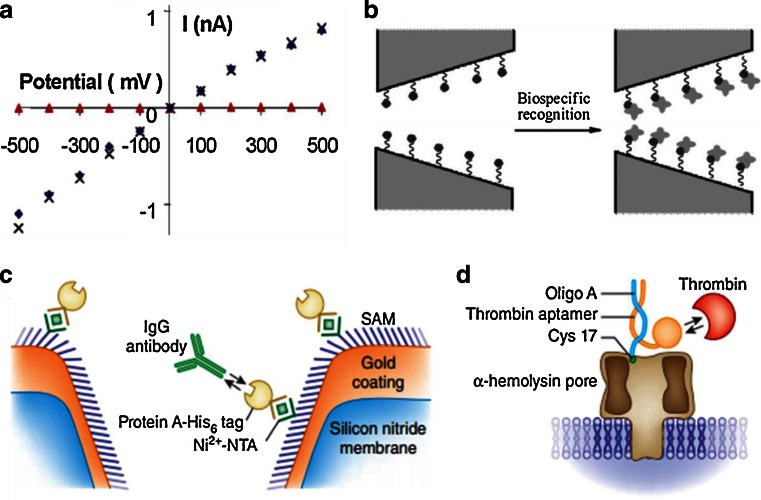
**a** Current–voltage curves for the streptavidin sensor are presented in the presence of no protein (×), 100 nM lysozyme (♦), and 180 pM SA (▲) [49]. Copyright © 2005, American Chemical Society **b** A single asymmetric nanopore functionalized with biorecognition elements (biotin-PEO_3_-amine) is used for streptavidin analytics [51]. Copyright © 2008, American Chemical Society **c** Artificial nanopores are applied to analyze the antibodies. His_6_-tagged protein A is immobilized on the surface of trisNTA-modified nanopores. IgG antibodies are identified because of their specific interaction times with protein A receptors. The species and subclass the antibodies belong to can also be detected in the system [56]. **d** An α-hemolysin pore functioned by a DNA aptamer detects thrombin [57]. Copyright © 2012, Rights Managed by Nature Publishing Group

The NPA technology develops in an application for detection of protein [46–48]. Siwy et al. fabricate a single conical gold nanopore with biochemical molecular-recognition agent (MRA) as a protein biosensor [49]. They investigate three MRA/analyte systems including the biotin/streptavidin, protein-G/immunoglobulin (IgG), and an antibody to the protein ricin as the MRA and ricin as the analyte. For example the diameter of the streptavidin (SA) molecule (~5 nm), and the final diameter after the MRA modified is ~5 nm for SA sensor. Because the size of protein molecule is comparable with the nanopore diameters, when the protein is recognized, the effective diameter of nanopore decreases. This is the signal off system. Current–voltage (I–V) curves for the biotinylated nanopore after exposure to two negative control proteins 100 nM lysozyme and BSA, the signal show little change, indicating that the sensor does not respond to proteins that do not recognize by the biotin MRA. In contrast, the ionic current drop substantially after immersed to a solution with 180 pM streptavidin (Fig. 2a). They use the time required for blockage, τ _b_, to determine the analyte concentration. The IgG concentrations in 100–10 nM range can be detected. Chen group also use the MRA to detect a variety of biotin binding proteins by modified an OmpG nanopore with a biotinylated PEG molecule [50].

Ali et al. report a facile strategy based on the electrostatically assemble biorecognition with the target into conical nanopores for constructing a nanobiosensor. In this system the target SA (∼5 nm) and the dimension of the nanopore (∼8 nm) are comparable in size and the detection limit of SA is 1 pM (Fig. 2b) [51–53]. Maglia et al. evolve Cytolysin A nanopore, which can be isolated into three nanopore types. The three ClyA nanopores with different diameter (33, 37, 42 Å) allowed the selective entry of proteins inside the nanopore [54, 55].

Recently, Wei et al. [56] detect single protein by using solid-state artificial nanopores modified with biological receptors (Fig. 2c). In their design, gold-coated silicon nitride nanopores functionalized with adequately few multivalent nitrilotriacetic acid (NTA) groups. By adjusting the ratio of NTA and the fabrication of ethylene glycol which can couple to NTA, only a single NTA tag can be controlled to fabricate on the nanopore. They use the NTA receptor, which act as binding sites for His-tagged proteins. When nanopores are immersed in the solution with His-tagged protein A, the current across it changes from an empty pore to a pore transiently blocked by one protein A molecule, or back-up process. The authors design another nanopore platform that is used to detect IgG. In this system, His_6_-tagged protein A is stably immobilized within a trisNTA-modified pore. IgG antibodies (=ligands) interact with the His_6_-tagged protein A, are detected by the resistive pulse technique. This nanopore sensor which based on how long the pore is blocked could detect single molecules of IgG and distinguish between various IgG subtypes from different organisms. Unbinding of His-tagged protein A was not detected. To control the concentration and geometry of receptors, Rotem et al. [57] also achieve a sensitivity detection of protein by using a α-hemolysin pore (Fig. 2d). Other groups use nanopores to study protein translation [58, 59], DNA-protein interactions [60, 61], and proteins folding [62, 63].

The NPA for detection of proteins has traits in common. (1) The nanopore mouth dimension and the target are comparable in size. (2) By choosing or tuning the receptors which can recognize or bind the specific proteins leads to the detection with high specificity. (3) When the proteins enter into the nanopores, combine with the receptor, lead to the decrease of the effective diameter, and the ion current decreases. These are signal off systems. NPA application in detection of protein can achieve single-molecule sensitivity because the diameter of nanopore can be controlled accurately. Compared with other certain protein detection technologies, the NPA offers a simple detection method relying on the electrical read-out to downstream signal processing, which avoiding complex operations.

### Detection of nucleic acids

**Fig. 3:**
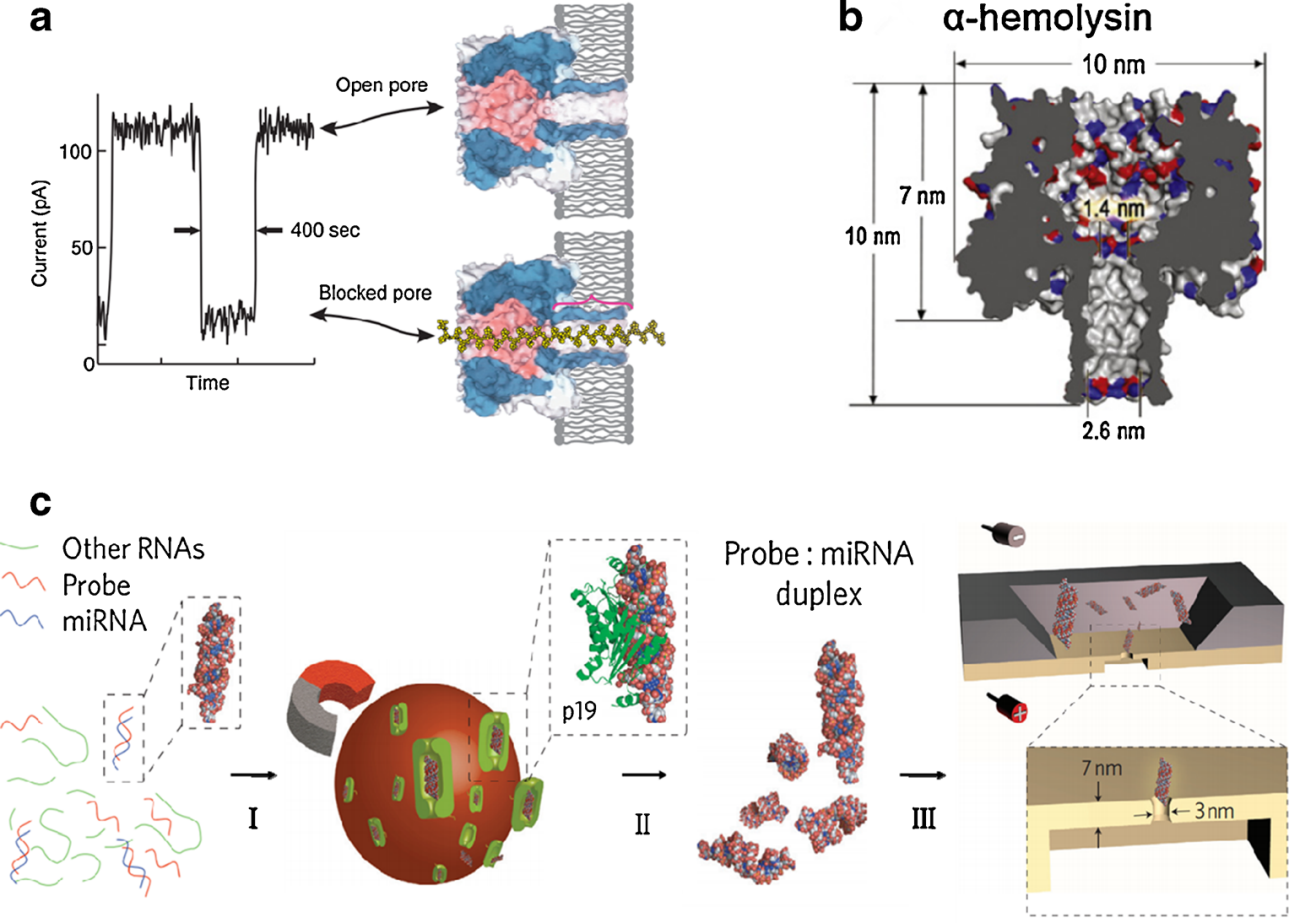
**a** The nanopore is used in strand-sequencing detection. The ionic current amplitude through a α-hemolysin nanopore changes in the two statuses, an open pore and a blocked one by a single-strand DNA. The red bracket means this system cannot distinguish the ~12 nucleotides [67]. Copyright © 2008, Rights Managed by Nature Publishing Group **b** Side view of α-hemolysin pore from Staphylococcus aureus is presented [69]. Copyright © 1996, American Association for the Advancement of Science **c** The specific miRNA is detected using solid-state 3-nm-diameter nanopore in a 7-nm-thick membrane [82]. Copyright © 2010, Nature Publishing Group

The NPA is firstly applied possibility to rapidly sequencing DNA [64–66]. In 1996 year, a group of scientists made a discovery that the ion channel could in principle provide direct, high-speed detection of the sequence of bases in single molecules of DNA or RNA [41]. They use an electric field to drive single-stranded DNA and RNA molecules through a pore-forming protein and detect the signal of ionic current in nanopores [67, 68] (Fig. 3a). This system uses the Staphylococcus aureus toxin, α-hemolysin (α-HL) (Fig. 3b), the use of which as a biosensor is pioneered by Bayley and his coworkers [69–73]. Both Ghadiri group and Akeson group show that polymerase enzymes can be used to move DNA across the α-HL nanopores [74–76]. Gundlach group introduced the MspA pore and showed the convincing sequencing data [77, 78]. Maglia et al. use modified ClyA nanopore to recognize and chaperone DNA [79]. Long group modified the protein nanopores as biomolecular sensors [80–83]. These experiments dawn some conclusions. For example, Contrast on polyC, polyA can block the nanopore a greater degree; the nanopore has not a clear distinct between purine and pyrimidine ribonucleotides. The order of the nucleobases in a polynucleotide can be detected by the signal changes of ion current though nanopores. Both kilo-base length polymers (single-stranded genomic DNA or RNA) and small molecules (e.g., nucleosides) can be identified and characterized without amplification or labeling. The NPA offers a unique analytical capability that makes inexpensive, rapid DNA sequencing possible [84, 85].

Artificial nanopores synthesized by materials such as silicon nitride have superiority to be detected of molecules. The capabilities of these sensors are influenced by both the diameter of the nanopore and the thickness of the membrane. Wanunu et al. show a sub-micrometer area of a SiN membrane with thicknesses as small as 6 nm provides a detection platform for the RNA molecules, which without the time-consuming labelling or amplification methods [86]. Their experiments show that reducing the thickness of the membrane can increase signal amplitudes from biomolecules. Nucleic acids with as few as ten base pairs can be detected by using 3-nm-diameter nanopores in sub-10-nm thick membranes. The various short nucleic acids with similar molecular weights are also discriminated on differences in their physical dimensions. They detect the miR122a which from 1 mg of rat liver total RNA using by a 3-nm-diameter nanopore in 7-nm-thick membrane. Target microRNA is first hybridized to a probe. This probe/target duplex is then enriched through binding to the viral protein. From the measurement data, the concentration of miR122a dilute 20-fold in sample is 0.7 fmol/μL, translate 78 ± 2 pg miR122a/mg liver RNA in rat liver cells. Gu et al. use one nanopore to detect of multiple miRNA simultaneously [87]. When it comes to the thickness of nanopore, the graphene nanopores have to mentioned because of it has one atom thickness. Graphene nanopores show great potential for the detection of DNA sequencing rather than other solid-state artificial nanopores [88–98]. The nanopore provides a highly confined space which made the nucleic acids analyzed at high throughput [99–101]. However, such thin membranes significantly limit the surface of the pore available to interact with DNA, leading to the translocation speeds of DNA in nanopores is too fast. Thus, electrical signal cannot be resolved with sufficient accuracy in the detection system [102, 103].

To regulate the translocation speeds of nucleic acid might be one of the most important factors in detection of DNA or RNA sequencing use solid-state artificial nanopores. During detection of nucleic acids sequencing both signals off system (nucleic acids enter into nanopores) and signal on system (nucleic acids come out of nanopores) are used together. Compared with the proteins, the size of nucleic acids are smaller which requires smaller and special character of nanopores, such as protein nanopores and graphene nanopores. Furthermore, taking advantage of nucleic acids are designable and modified, the NPA can combined with conventional characterization techniques such as gel electrophoresis, atomic force microscopy, transmission electron microscopy and laser scanning confocal microscopy. The amplification method such as the interaction with protein can improve the detection limit of nucleic acids by the NPA technology.

### Detection of small molecules

**Fig. 4:**
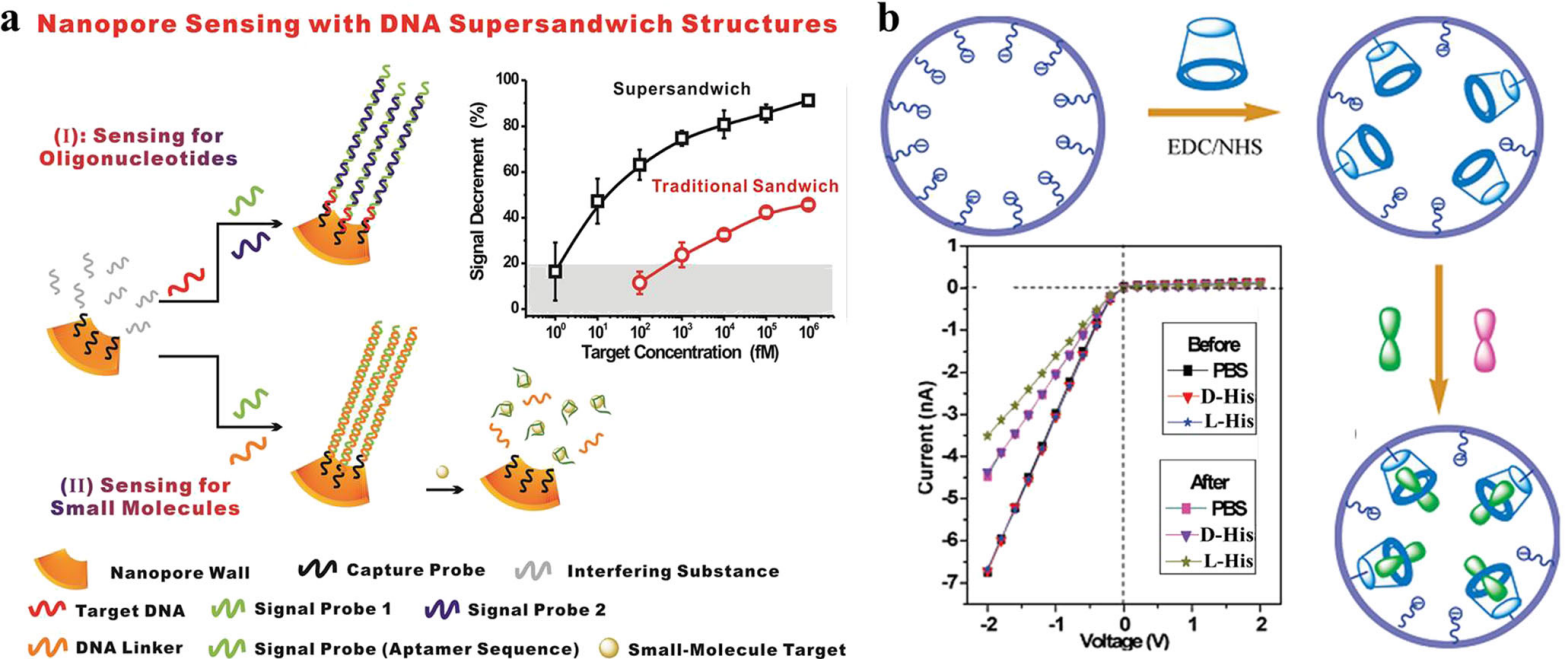
**a** The scheme of two-way nanopore sensor. A more complex DNA nanostructure (supersandwich structures) can be introduced to the nanopore. It shows high sensitive to DNA or ATP. The detection limt of DNA is 10 fM and ATP is 1 nM. More importantly, it could also be used in complex matrices when the interfer substances concentration is high in the buffer solution. It even can be directly used in serum [31]. Copyright © 2013 WILEY-VCH Verlag GmbH & Co. KGaA, Weinheim **b** The scheme of β-CD modified nanopore system. It is responsive to L-His and induce a large difference in the enantiomeric ionic currents which makes it can be used in practical application [108]. Copyright © 2011, American Chemical Society

The NPA technology can be used to detect and select small molecules by binding them in nanopores. Bayley H et al. equipped α- hemolysin nanopore with cyclodextrins to detect of organic molecules [104]. Kasianowicz and Bezrukov group studied the interaction between polymer molecules and protein nanopores [105–107]. Both of them examine the blockages of ion current when analysts through nanopores, which belongs to signal off system. Till now, small molecules and nucleic acids cannot be analyzed simultaneously in a nanopore sensor. Xia et al. have found that a more complex DNA nanostructure can be introduced to the nanopore [31]. The complex DNA nanostructure contains multiple target-binding sites on each of its long concatamers and provides a built-in amplification mechanism (Fig. 4a). This nanopore is prepared from poly(ethyleneterephthalate) membranes. It has a diameter of 79 ± 7 nm. When target nucleic acids exist, the DNA supersandwich structures are assembled to decrease the effective diameter of the nanopore; while ATP exists, the DNA supersandwich structures are disassembled to increase the effective diameter of the nanopore, that means both the signal off and signal on system in this nanopore detection platform. This nanopore sensor has many advantages, such as enhancing signal intensity, a better detection limit (the detection limit of DNA is 10 fM and ATP is 1 nM), and anti-interference capability. More importantly, it can be used to analysis single-base mismatch resolution and discrimination among different types of nucleoside triphosphates. It could also be used in complex matrices when the interfering substances concentration is high in the buffer solution. It even can be directly used in serum.

Li et al. report a simple enantioselective device. They modify a single artificial β-cyclodextrin (β-CD) to the single conical nanopore system (Fig. 4b) [108]. Putting this β-CD-modified nanopore into a solution of L-His, they find a decrease of the transmembrane ionic current due to the selective binding of L-His to the nanopore wall that is occurs inside the confined geometry. While, immersing this β-CD-modified nanopore in solutions of D-His or other aromatic amino acids, no significant changes of ionic current are found. It shows that this β-CD-modified nanopore has high selective recognition of histidine enantiomers by monitoring of ionic current signatures. They also find that the ionic currents decrease gradually with increasing L-His concentration from 0 to 1 mM.

There are some characteristics of the NPA using in the detection of small molecules. (1) With the size of small molecules down, the detection by binding event direct which require the smaller diameter of nanopores (most of protein nanopores); (2) Detecting of small molecules using the NPA, the signal changes are inconspicuous without the amplified technology. (3) Statistical analysis of each blockage should be investigated in nanopore experiments [109]. (4) The NPA detection platform is expected to develop into a live assay for disease related molecular targets, and with many practical applications in biotechnology and life science.

### Detection of ions

**Fig. 5:**
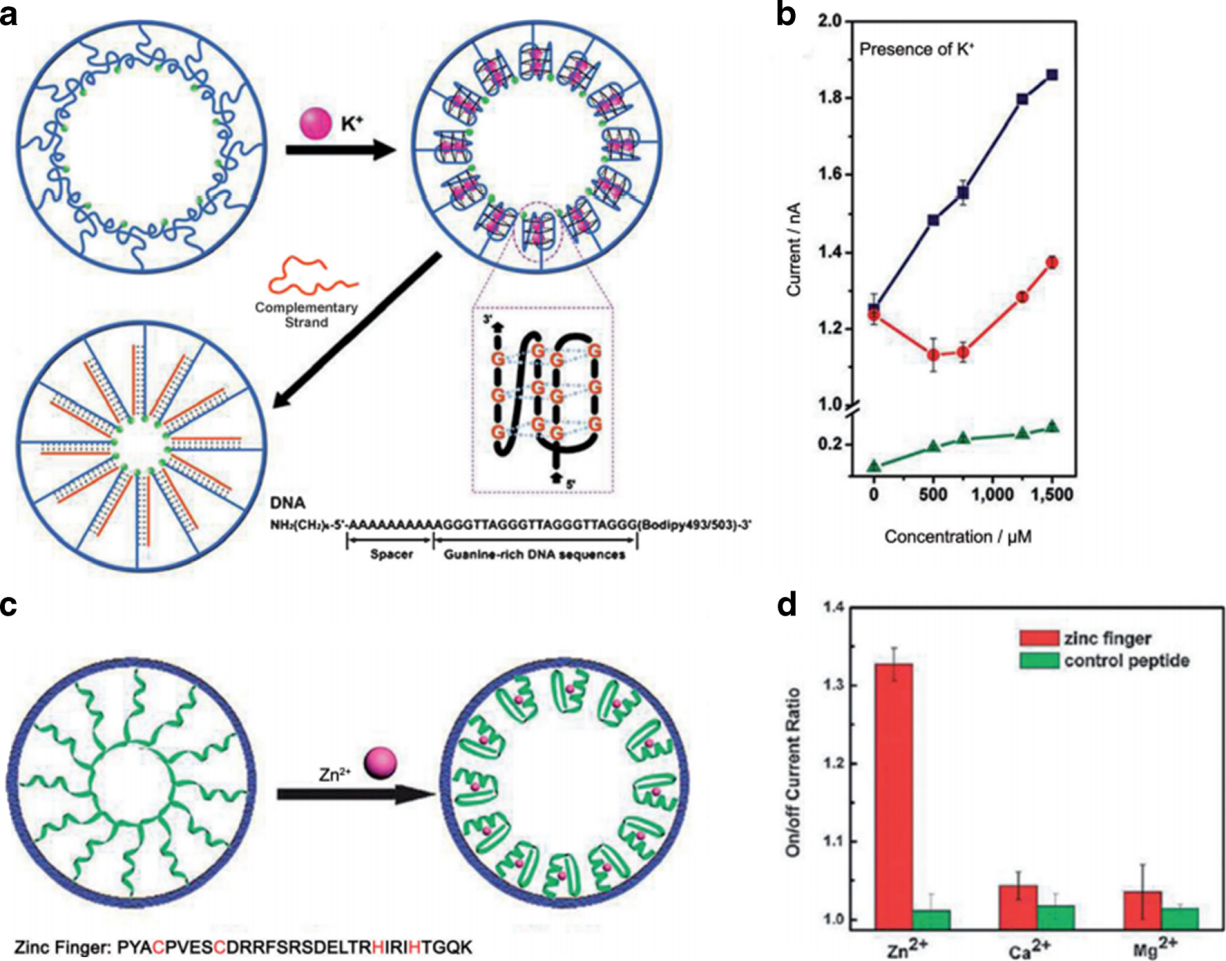
**a** A single nanopore is fabricated by conformational transition of G4 DNA which can response to K^+^ [110]. Copyright © 2009 American Chemical Society **b** The current data of the NPA system. The blue line is the absence of G4 DNA modification. The red line is the presence of G4 DNA modification. The green line is the addition of the complementary DNA strands. **c** The scheme of biomimetic zinc activated ion channel. **d** The histogram of the nanopore. It shows the specificity of the system [111]. Copyright © 2010 Royal Society of Chemistry

Jiang et al. report a potassium-responsive nanopore (Fig. 5a) [110]. It is mainly rely on the conformational changes of the G4 DNA chains in the presence of potassium ions (K^+^). The reason is that the structure transition of the G4 DNA chains from loose packing to the i-motif structure after binding with K^+^. Thus the effective pore sizes decrease which leading to the ionic current drop. As shown in Fig. 5b, this nanopore/DNA hybrid system has an ion concentration effect that provides a nonlinear response to K^+^ at the concentration ranging from 0 to 1500 μM. They also construct a biomimetic zinc activated ion channel by introducing the zinc fingers into the nanopores (Fig. 5c) [111]. This nanopore is responsive to zinc ions. In the presence of zinc ions, the zinc fingers fold into finger like conformations, thus the effective diameter of the channel increase. In turn, the biomimetic ion channel is activated. However, in the absence of zinc, they find a low ion conductance. This nanopore has high specificity. As shown in Fig. 5d, it is not responsive to other metal ions. If introduce a T-rich ssDNA to the nanopores, it can construct a biomimetic mercury (II)-gated nanopore by forming a stable T-Hg^2+^-T complex [112]. Cu^2+^ also is detected by peptide conformational changes in nanopores [113].

The dimension of ions is the smallest among targets, which means that they can only be detected by indirect analysis, such as the interactions with nanopores, the conformation transition between ions and the nucleic acids or polypeptide et al. The conformational change induces the change in the effective size of nanopores and these are either signal on or signal off system. The key features of the NPA using for ions detection is closely imitate these molecular interactions happening in living organisms.

## Summary

The advantages of the NPA for detection of biochemical species are as follows:

1. The NPA has high sensitivity; even the single molecule can be detected.
2. The NPA has high specificity; the target can be detected from the analogues or in the presence of interfering substance.
3. The NPA for target detection requires very low sample volumes and without sample complicated preparation.
4. Study the dynamics of interaction between receptors and targets in nanopores by the patch clamp technique can promote the understanding of the molecular mechanism.
5. The NPA has the broad analytical range. In addition to the biomolecules, small molecules and ions, other molecules such as nanoparticles [114], inorganic molecules [115–117] and organic molecules [118] can also be detected.

There are also many challenges in application the NPA for detection of biochemical species.

1. The limit of materials and dimensions cause that the NPA for detection platform can only be used in vitro.
2. How to further improve the NPA technology for detection of targets specificity. Especially for proteins which have complex spacial structures, the specificity is even more important in bimolecular analysis.

Considered that with the development of nanotechnique, the fabrication of nanopores will be more diverse and accurate. The NPA as a new kind of detection methods arouses widespread interest for its incredibly merits and wide application. It presents improved capabilities for the area of single molecule detection, discriminating molecules with different configuration and mimicking the transmembrane protein features. The NPA is expected that the simple electronic device fabricated with high sensitivity and specificity, which can be used in practical life, such as clinic diagnostics, routine laboratory detection, food safety, and environmental monitoring.
